# Rapid and inexpensive body fluid identification by RNA profiling-based multiplex High Resolution Melt (HRM) analysis

**DOI:** 10.12688/f1000research.2-281.v2

**Published:** 2014-02-26

**Authors:** Erin K. Hanson, Jack Ballantyne

**Affiliations:** 1 National Center for Forensic Science, Orlando, FL 32816-2367, USA; 2 Department of Chemistry, University of Central Florida, Orlando, FL 32816-2366, USA

**Keywords:** Forensic Science, Body Fluid Identification, Messenger RNA (mRNA) Profiling, High Resolution Melt (HRM) Analysis

## Abstract

Positive identification of the nature of biological material present on evidentiary items can be crucial for understanding the circumstances surrounding a crime. However, traditional protein-based methods do not permit the identification of all body fluids and tissues, and thus molecular based strategies for the conclusive identification of all forensically relevant biological fluids and tissues need to be developed. Messenger RNA (mRNA) profiling is an example of such a molecular-based approach. Current mRNA body fluid identification assays involve capillary electrophoresis (CE) or quantitative RT-PCR (qRT-PCR) platforms, each with its own limitations. Both platforms require the use of expensive fluorescently labeled primers or probes. CE-based assays require separate amplification and detection steps thus increasing the analysis time. For qRT-PCR assays, only 3-4 markers can be included in a single reaction since each requires a different fluorescent dye.

To simplify mRNA profiling assays, and reduce the time and cost of analysis, we have developed single- and multiplex body fluid High Resolution Melt (HRM) assays for the identification of common forensically relevant biological fluids and tissues. The incorporated biomarkers include IL19 (vaginal secretions), IL1F7 (skin), ALAS2 (blood), MMP10 (menstrual blood), HTN3 (saliva) and TGM4 (semen).  The HRM assays require only unlabeled PCR primers and a single saturating intercalating fluorescent dye (Eva Green). Each body-fluid-specific marker can easily be identified by the presence of a distinct melt peak. Usually, HRM assays are used to detect variants or isoforms for a single gene target. However, we have uniquely developed duplex and triplex HRM assays to permit the simultaneous detection of multiple targets per reaction. Here we describe the development and initial performance evaluation of the developed HRM assays. The results demonstrate the potential use of HRM assays for rapid, and relatively inexpensive, screening of biological evidence.

## Introduction

Identification of the tissue source of biological material present on individuals, evidentiary items and at crime scenes can be crucial to a fuller understanding of the circumstances pertaining to a crime. However traditional protein-based body fluid identification methods use a variety of labor intensive and technologically diverse techniques that do not permit the identification of all body fluids and tissues. Thus there remains a need to develop definitive molecular based strategies for the conclusive identification of all forensically relevant biological fluids and tissues. Although protein ^1– 3^, epigenetic DNA markers ^4– 10^ and microRNAs ^11– 17^ are promising examples of such a molecular based approach, messenger RNA (mRNA) profiling represents the current gold standard in this area due to the identification and development of a number of specific and sensitive mRNA assays for the identification of all of the forensically-relevant body fluids and tissues, namely blood, semen, saliva, vaginal secretions, menstrual blood and skin ^13, 18– 36^. Importantly, messenger RNA markers are surprisingly stable in the dried state in that they can be successfully detected in environmentally impacted and partially degraded samples ^20, 22, 25, 26, 37– 40^.

Current mRNA body fluid identification assays typically involve capillary electrophoresis (CE) or quantitative RT-PCR (qRT-PCR) platforms ^13, 20, 24, 28, 29, 31, 32, 34, 35^, each with its own limitations. Both platforms require the use of expensive fluorescently labeled primers or probes. CE-based assays require separate amplification and detection steps thus increasing the time required for analysis. For qRT-PCR assays, only 3 or 4 markers can be included in a single reaction since each marker requires a different fluorescent dye.

In an attempt to simplify mRNA profiling assays, and to reduce the time and cost of analysis, we have investigated alternative technology solutions for mRNA profiling, particularly High Resolution Melt analysis (HRM). HRM is a rapid and relatively cheap bio-analytical method that was initially developed for sequence variant screening (i.e. mutation analysis) but which has also found utility in single nuclear polymorphism (SNP) typing ^41– 44^, methylation analysis ^45, 46^, copy number variant confirmation ^47– 49^, clone characterization ^50^ and as an alternative for gel electrophoresis ^51^. As its name suggests, HRM is a technique that permits the identification of specific PCR products (i.e. amplicons) by their melting temperature ( *T*_m_). An amplicon’s precise melting temperature is dependent on its sequence, length and the ionic strength of its environment and can be measured post-PCR to within 0.1°C with specialized software and hardware. HRM assays require only the use of unlabeled PCR primers and a single saturating intercalating fluorescent dye (e.g. Eva Green). The latter provides enhanced sensitivity by ensuring that all available double stranded DNA binding sites are saturated during the melting process. After amplification the amplicon is melted slowly by increasing the temperature and a loss of fluorescence occurs as the DNA strands separate and release the bound saturating dye into solution. By measuring the negative first derivative of fluorescence (F) with respect to temperature (T) (-dF/dT) a distinct and characteristic melt curve is obtained with the peak maximum representing the *T*_m_. Importantly, due to the high resolution nature of the amplicon melt analysis, it is possible to perform multiplex analysis of several amplicons in one tube ^52^.

Here we describe the development and initial performance evaluation of singleplex and multiplex RNA-based HRM assays for the identification of the commonly encountered forensically relevant body fluids and tissues.

## Methods

### Body fluid samples

Body fluids were collected from volunteers using procedures approved by the University’s Institutional Review Board. Informed written consent was obtained from each donor. Blood samples (10mL, Bioreclamation, Westbury, NY) (16 donors; male and female; 21–56 yrs old) were collected by venipuncture into additive-free vacutainers and 50 μl aliquots were placed onto cotton cloth and dried at room temperature. Freshly ejaculated semen (12 donors; 30–55 yrs old) was provided in sealed plastic tubes and stored frozen. After thawing, the semen was absorbed onto sterile cotton swabs and allowed to dry. Buccal samples (saliva) (18 donors; male and female; 26–60 yrs old) were collected from donors using sterile swabs by swabbing the inside of the donor’s mouth. Semen-free vaginal secretions (18 donors; 20–60 yrs old) and menstrual blood (5 donors; 20–33 yrs old) were collected using sterile cotton swabs. Human skin total RNA was obtained from commercial sources: Stratagene/Agilent Technologies (Santa Clara, CA), Biochain ^®^ (Hayward, CA), Zenbio (Research Triangle Park, NC), and Zyagen (San Diego, CA). Cellular skin samples were collected by swabbing human skin or a touched object surface with a sterile water pre-moistened sterile swab. All samples were stored at -20°C or at room temperature until needed. A 50 μl stain or a single cotton swab was used for RNA isolation.

### RNA isolation and quantitation

Total RNA was extracted from blood, semen, saliva, vaginal secretions, menstrual blood and skin using a manual organic RNA extraction (guanidine isothiocyanate-phenol-chloroform mixture) ^28– 32^. Briefly, 500 μl of pre-heated (56°C for 10 minutes) denaturing solution (4 M guanidine isothiocyanate, 0.02 M sodium citrate, 0.5% sarkosyl, 0.1 M β-mercaptoethanol) was added to a 1.5 mL Safe Lock tube extraction tube (Eppendorf, Westbury, NY) containing the stain or swab. The samples were incubated at 56°C for 30 minutes. The swab or stain pieces were then placed into a DNA IQ ^TM^ spin basket (Promega, Madison, WI), re-inserted back into the original extraction tube, and centrifuged at 14,000 rpm (16,000 × g) for 5 minutes. After centrifugation, the basket with swab/stain pieces was discarded. To each extract the following was added: 50 μl 2 M sodium acetate and 600 μl acid phenol:chloroform (5:1), pH 4.5 (Ambion by Life Technologies). The samples were placed at 4°C for 30 minutes to separate the layers and then centrifuged for 20 minutes at 14,000 rpm (16,000 × g). The RNA-containing top aqueous layer was transferred to a new 1.5 ml microcentrifuge tube, to which 2 μl of GlycoBlue™ glycogen carrier (Ambion by Life Technologies) and 500 μl of isopropanol were added. RNA was precipitated for 1 hour at -20°C. The extracts were then centrifuged at 14,000 rpm (16,000 × g). The supernatant was removed and the pellet was washed with 900 μl of 75% ethanol/25% DEPC-treated water. Following a centrifugation for 10 minutes at 14,000 rpm (16,000 × g), the supernatant was removed and the pellet dried using vacuum centrifugation (56°C) for 3 minutes. Twenty microliters of pre-heated (60°C for 5 minutes) nuclease free water (Ambion by Life Technologies) was added to each sample followed by an incubation at 60°C for 10 minutes. Samples were used immediately or stored at -20°C until needed. All extracts were DNase treated to remove residual DNA using the Turbo DNA- *free*™ kit (Applied Biosystems (AB) by Life Technologies, Carlsbad, CA) according to the manufacturer’s protocol. RNA extracts were quantitated with Quant-iT™ RiboGreen ^®^ RNA Kit (Invitrogen by Life Technologies, Carlsbad, CA) as previously described ^28– 32^. Fluorescence was determined using a Synergy™ 2 Multi-Mode microplate reader (BioTek ^®^ Instruments, Inc., Winooski, VT).

### cDNA synthesis

All samples were reverse transcribed using the High Capacity cDNA Reverse Transcription kit (AB by Life Technologies) according to manufacturer’s protocols. The desired total RNA input was reverse transcribed in a 20 μl RT reaction volume (standard input – 25 ng total RNA). If no quantitative value was obtained or quantitation was not performed, an aliquot of the total RNA extract was used (14.2 μl). A reverse transcription negative reaction (containing total RNA and reaction buffer but no reverse transcriptase enzyme) was performed for each sample.

### High Resolution Melt (HRM) analysis

Singleplex, Duplex, Triplex Assays: HRM assays were performed using the Type-It ^®^ HRM™ PCR kit (QIAGEN, Germantown, MD). Each 25 μl reaction included: 1 × HRM master mix (contains HotStar Taq Plus DNA Polymerase, EvaGreen dye, and manufacturer-designated ‘optimized concentrations’ of Q-solution (commercial reagent, exact concentrations not specified by the manufacturer), dNTPs and MgCl _2_), 2.8 μM primers ( Table 1), 2 μl of RT reaction (cDNA), and RNase-free water. All assays were run on the Rotor-Gene ^®^ Q real time PCR instrument (QIAGEN), using the following cycling conditions: 95°C 5 min, followed by 45 cycles of 94°C 10 sec, 55°C 30 sec, 72°C 10 sec. HRM analysis was performed using a 65–90°C temperature range, with +0.1°C increments (90 sec of pre-melt conditioning on first step; and 2 sec for each step afterwards).

**Table 1.** Genes and amplicons used in singleplex, duplex and triplex body fluid HRM assays.

Body fluid	Gene	Accession number	Amplicon size (bp)	Amplicon T _m_		Primer sequence (5′–3′)
				°C	Range (± 3SD)	
Vaginal	IL19	NM_153758	189	81.5	81.3 – 81.7	F: AACCACGGTCTCAGGAGATG R: GAACGCCAGGAGGTTCTTG
Skin	IL1F7	NM_014439	92	83.2	82.9 – 83.5	F: CCAGTGCTGCTTAGAAGACC R: TCACCTTTGGACTTGTGTGAA
Blood	ALAS2	NM_001037967	136	85.8	85.6 – 86.0	F: TGTGTCCGTCTGGTGTAGTA R: AAACTTACTGGTGCCTGAGA
Menstrual blood	MMP10	NM_002425	227	82.2, 83.5	81.8 – 82.7, 83.0 – 84.0	F: ACAGGGAAGCTAGACACTGA R: CTGGAGAATGTGAGTGGAGT
Saliva	HTN3	NM_000200	134	76.3	75.9 – 76.7	F: GCAAAGAGACATCATGGGTA R: GCCAGTCAAACCTCCATAATC
Semen	TGM4	NM_003241	164	82.3	82.2 – 82.4	F: ATGGTGTAAAGAGGACATGGTT R: GGGAAATGCAGCAGTCCAG

‘All Body Fluids’ Hexaplex Assay: HRM assays were performed using the Type-It ^®^ HRM™ PCR kit (QIAGEN, Germantown, MD). Each 25 μl reaction included: 1X HRM master mix (contains HotStar Taq Plus DNA Polymerase, EvaGreen dye, and optimized concentrations (commercial reagent, exact concentrations not specified by the manufacturer) of Q-solution, dNTPs and MgCl _2_), 0.56 – 1.4 μM primers ( Table 3) (ALAS2 – 0.56 μM, TGM4 – 0.56 μM, HTN3 – 1.4 μM, IL19 – 0.84 μM, MMP10 – 0.56 μM, CCL27 – 0.56 μM), 2 μl of RT reaction (cDNA), and RNase-free water. All assays were run on the Rotor-Gene ^®^ Q real time PCR instrument (QIAGEN), using the following cycling conditions: 95°C 5 min, followed by 45 cycles of 94°C 10 sec, 57°C 40 sec, 72°C 20 sec. HRM analysis was performed using a 73–90°C temperature range, with +0.1°C increments (90 sec of pre-melt conditioning on first step; 2 seconds for each step afterwards).

## Results

### Duplex HRM assays for body fluid identification

**Figure 1.**
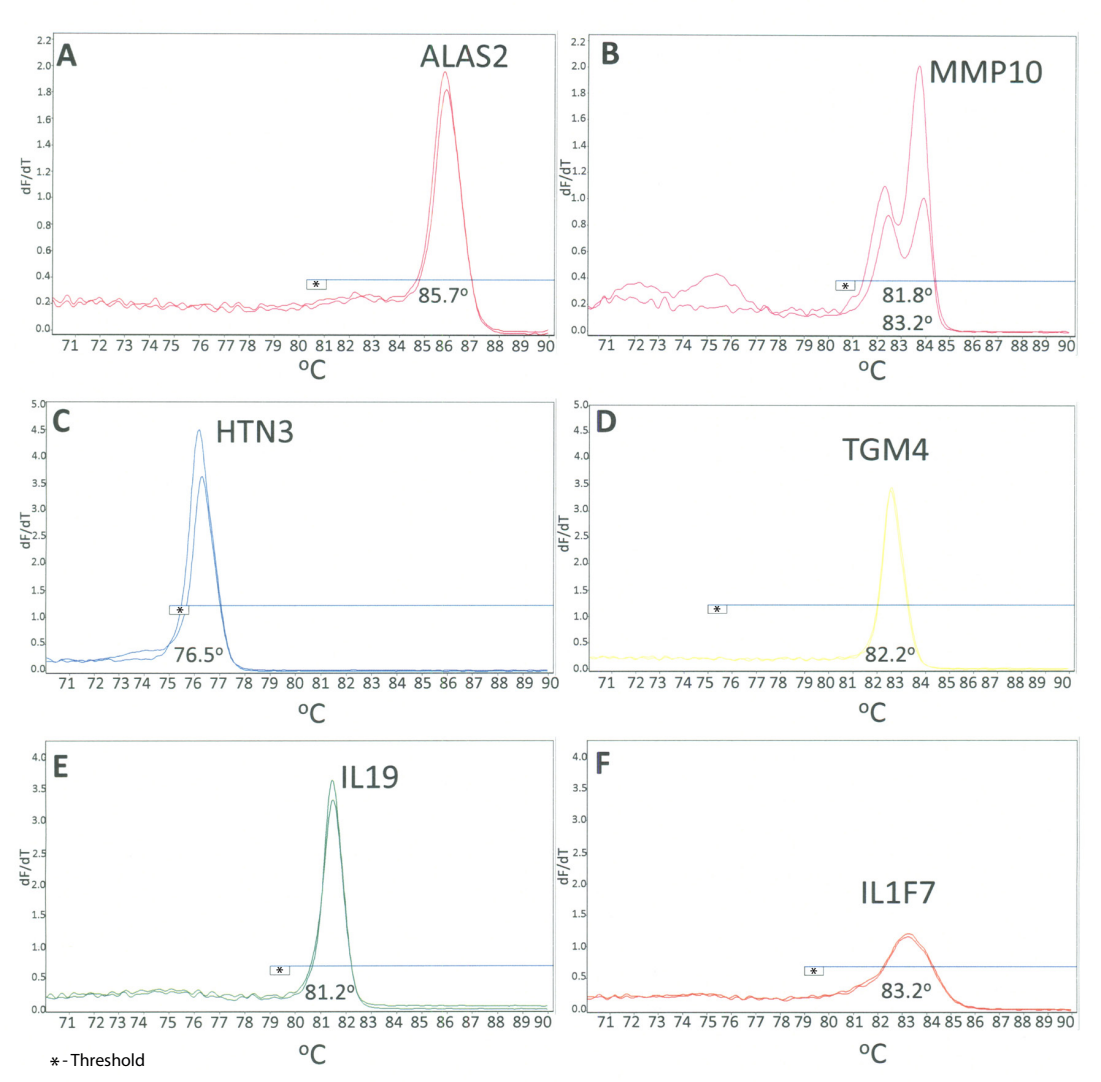
Singleplex HRM body fluid assays. High resolution derivative melt curve plots for individual body fluid specific markers: **A**) ALAS2 (blood); **B**) MMP10 (menstrual blood); **C**) HTN3 (saliva); **D**) TGM4 (semen); **E**) IL19 (vaginal secretions); **F**) IL1F7 (skin). The plots from two different donors are shown for each marker. The x-axis represents the temperature and the y-axis indicates the first derivative of the change of fluorescence with temperature (-dF/dT). The average *T*_m_ (°C) for each marker is displayed. The horizontal line on each plot represents the analysis threshold.

**Figure S1.**
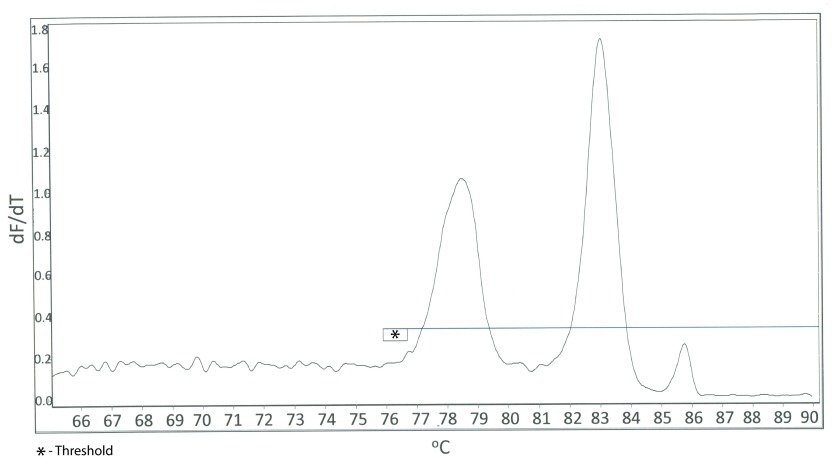
DNA products observed for MMP10 Singleplex HRM Assay. High resolution derivative melt curve plot for a DNA sample for the MMP10 singleplex HRM assay. The presence of two products (78.5 and 83.1°C) was observed. The x-axis represents temperature (°C) and the y-axis represents the first derivative of the change of fluorescence with temperature (-dF/dT). The horizontal line on each plot represents the analysis threshold.

***Development of the HRM assay*.** Based on our previous work with mRNA profiling ^13, 24, 28, 29, 31, 32^, we selected one marker for each body fluid/tissue based on amplification efficiency, specificity and/or sensitivity. The fluid- and tissue-specific markers selected were ALAS2 (blood) ^21, 22, 32^, MMP10 (menstrual blood) ^32^, HTN3 (saliva) ^26, 30– 32^, TGM4 (semen) ^26, 53^, IL19 (vaginal secretions) ^29^ and IL1F7 (skin) ^27, 28^ ( Table 1). We performed singleplex HRM assays for each of these markers to ensure that they could be uniquely identified based on their different *T*_m_s. Samples from two donors of each body fluid of interest were used to estimate the *T*_m_ of each of the selected markers. The observed *T*_m_s are listed in Table 1 and the melt curves for each marker are displayed in Figure 1. Derivative plots are shown, with the temperature range (70–90°C) used in the HRM assay is displayed along the x-axis and the -dF/dT value (negative first derivative of fluorescence (F) with respect to temperature (T)) is displayed along the y-axis. An analysis threshold can be set for each axis, which is represented by a horizontal (blue) line on each plot. As can be seen in Figure 1, a single melt curve was observed for most markers with the exception of MMP10 in which two products are observed (81.8°C and 83.2°C). The same primer sequences for MMP10 used in these experiments are also used in our CE-based mRNA profiling multiplexes, where only one product is observed. Upon evaluation of the amplified sequence and comparison to different MMP genes, it was determined that the MMP10 primer set was capable of also amplifying the MMP3 gene. There are four mismatched bases in the forward primer, three of which are in the 3´end of the primer (i.e. 3 mismatches in the last 9 bases of the primer). This likely therefore causes amplification inefficiencies and results in a failure to detect the MMP3 product in some assays. The standard HRM protocol (QIAGEN Type-It ^®^ HRM™ kit) utilizes a 55°C annealing temperature which may explain why this second product is detected using this platform. Additional work would be needed to conclusively determine if this second peak is in fact MMP3 and to determine if the primer sequences can be modified to be MMP10-specific. MMP10 is also the only marker in the body fluid set in which HRM DNA products were observed, one at 78.5°C and another at 83.1°C ( Supplementary Figure 1). These were clearly distinguishable from a positive mRNA MMP10 result (82.2°C and 83.5°C). The presence of two DNA products further supports the hypothesis that MMP3 is being co-amplified, one DNA product resulting for MMP3 and MMP10 (expected DNA product size is 319 and 369 bp, respectively).

**Figure 2.**
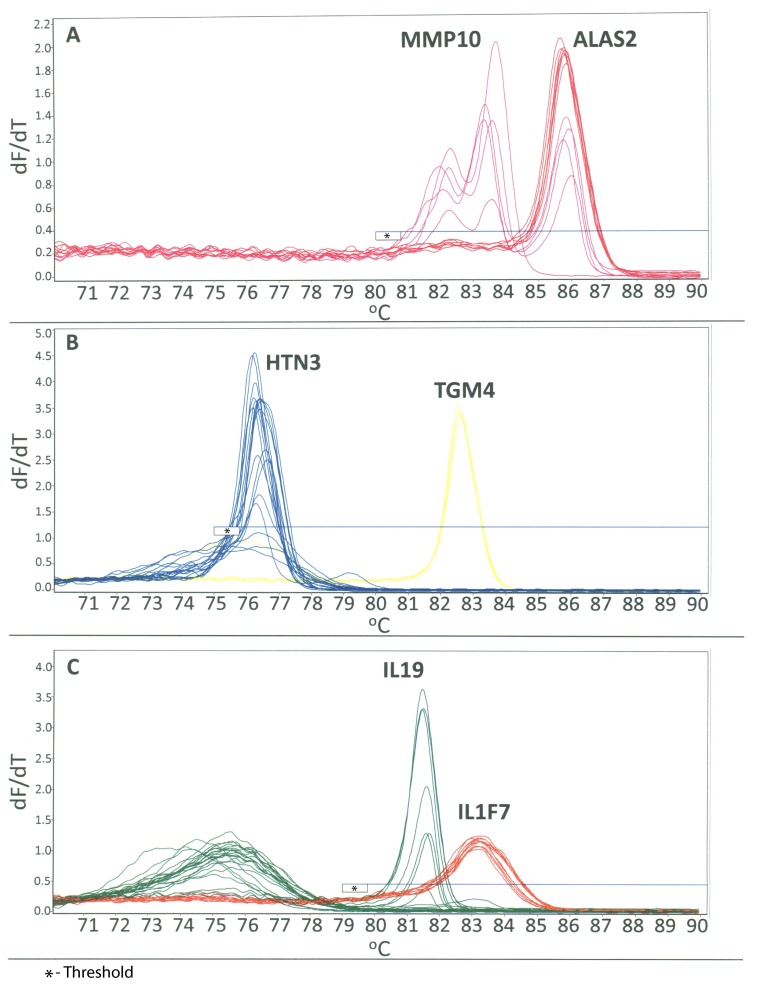
Duplex HRM body fluid assays. Duplex HRM assays incorporate a body fluid specific marker for each of two body fluids or tissues into a single reaction. High resolution derivative melt curve plots for the duplex assays are shown (where n = number of biological replicates (i.e. different individuals)): **A**) blood/menstrual blood (respectively: ALAS2, red, n = 8; MMP10, pink, n = 5); **B**) saliva/semen (respectively: HTN3, blue, n = 18; TGM4, yellow, n = 5); **C**) vaginal secretions/skin (respectively: IL19, green, n = 10; IL1F7, orange, n = 10). For interpretation of the reference to color, the reader is directed to the online version of the article.

While some of the observed *T*_m_s were similar or overlapping ( Table 1), there was sufficient resolution to incorporate all markers into three separate duplex reactions: blood/menstrual blood, semen/saliva and vaginal secretions/skin. For the development of the duplex HRM assays, the primer sets for each of the two markers were incorporated into a single reaction. No other parameters of the HRM assay were modified from the original singleplex assays. For each duplex HRM assay, known samples (total n = 56–65) of each of the targeted body fluids of interest were detected ( Figure 2, Table 2). The melt curves obtained during the initial testing of the duplex HRM assays are shown in Figure 2. The melt curves for each marker are shown overlaid in order to indicate their relative locations within the duplex. For each of these single source samples, only the expected *T*_m_(s) for the body fluid of interest was observed, with the exception of the menstrual blood samples ( Figure 2A). With respect to the latter, as can be seen from the “pink” melt curves (representing the known menstrual blood samples), both MMP10 and ALAS2 were detected in the majority of samples (4/5). This is expected, as menstrual blood samples will contain varying amounts of peripheral blood, which is demonstrated by the presence of ALAS2. We observe the same co-detection in CE based mRNA profiling assays ^31^. The simultaneous identification of both MMP10 and ALAS2 in individual menstrual blood samples also serves to confirm the functionality of this particular duplex assay.

**Table 2.** HRM duplex assay specificity. The number of samples from individuals in which the marker was successfully detected is displayed (numerator) out of the total number of individuals tested (denominator). The shading reflects the number of positive samples out of the total number tested: white – no detection; light grey 1–59%; dark grey ≥ 60%.

Body fluid/Tissue (25 ng RT)	Vaginal/Skin (n = 56)		Blood/Menstrual (n = 63)		Saliva/Semen (n = 65)	
	IL19	IL1F7	ALAS2	MMP10	HTN3	TGM4
Vaginal	**6/10**	**0/10**	**0/18**	**4/18**	**0/18**	**0/18**
Skin	**0/10**	**10/10**	**0/10**	**0/10**	**0/11**	**0/11**
Blood	**0/8**	**0/8**	**7/8**	**0/8**	**0/8**	**0/8**
Menstrual	**3/5**	**0/5**	**4/5**	**5/5**	**0/5**	**0/5**
Saliva	**0/18**	**0/18**	**0/18**	**0/18**	**15/18**	**0/18**
Semen	**0/5**	**0/5**	**0/4**	**0/4**	**0/5**	**4/5**

During the development of the duplex assays, we observed the appearance of broad peaks (“humps”) in the lower temperature ranges for the TGM4 and IL19 melt curves in the semen/saliva and vaginal/skin duplex assays, respectively ( Figure 2B and 2C). The artifacts were observed in the amplification blanks (data not shown) and are likely due to primer dimers or non-specific primer interactions. For the vaginal/skin assay, this does not interfere with data interpretation as the *T*_m_s of these markers are at a higher temperature. Since the HRM temperature range was kept consistent for all duplex assays, the x-axis threshold can simply be set to exclude the affected temperatures from analysis. However, for the semen/saliva assay, the artifacts originating from the TGM4 melt curves are located within the HTN3 *T*_m_ region. This could affect the interpretation of the data on this assay as it could appear as a positive HTN3 result, although the peaks are abnormally broad and are distinguishable from true peaks. The intensity of the saliva sample melt curves permits us to use a suitably high x-axis threshold in order to eliminate these “humps” from analysis.

### Specificity

A number of different donors (4–18 per body fluid or tissue, Table 2) of each body fluid were tested using all three duplex assays (total n = 56 (vaginal/skin), n = 63 (blood/menstrual blood, n = 65 (saliva/semen), Table 2). For the semen/saliva duplex, semen and saliva were correctly identified in a majority of samples and no cross-reactivity was observed with any of the other body fluids. For the vaginal/skin assay, vaginal secretions and skin were correctly identified in a majority of samples, and no cross-reactivity was observed for blood, semen or saliva ( Table 2). As can be seen from Table 2, 3/5 menstrual blood samples evaluated were positive for IL19 (vaginal secretions) which is not surprising since menstrual blood samples are expected to contain varying amounts of vaginal secretions. However, it does indicate the need for additional interpretation guidelines for menstrual blood. For the blood/menstrual blood assay, blood and menstrual blood was correctly identified in a majority of blood and menstrual blood samples and no cross-reactivity was observed with skin, saliva and semen. However, MMP10 (menstrual blood marker) was detected in ~20% of vaginal secretion samples (4/18). While early work with MMP10 demonstrated a high degree of specificity for menstrual blood, other studies have also reported detection of MMP10 in vaginal samples ^20^. Enzymes of the matrix metalloproteinase (MMP) family that play a role in the tissue remodeling that takes place during menstruation are spatiotemporally expressed in endometrial tissue ^54, 55^. Therefore, it is possible that increased levels of MMP10 may be present just prior to menses. The time during the reproductive cycle in which the blood samples in this study were collected was not known. Further work will be needed to evaluate MMP10 expression levels throughout the female reproductive cycle to determine if any trends can be identified amongst numerous donors.

A number of false negative results (9/56, 16%) were observed for the body fluids or tissues of interest for the various duplex assays ( Table 2). However, no false negatives were observed for skin (IL1F7 detected in all 10 donors tested) and menstrual blood (MMP10 detected in all 5 donors tested) ( Table 2). The precise reason for the absence of the body fluid specific markers in these known samples is unclear. The false negative results for vaginal secretions and saliva may be explained by possible differences in input quantity. All body fluid sample extracts are quantitated to allow for standard amounts of total RNA (i.e. 25 ng) to be added to each reverse transcription reaction. However, since no validated human specific RNA quantitation method is currently available, the quantitation values obtained for body fluids with commensal bacteria such as vaginal secretions and saliva may be inaccurate with respect to estimates of the amount of human RNA in such samples. While attempts were made to normalize total RNA input into these body fluid samples, the actual amount of total human RNA may be somewhat less. This could lead to false negative results since sufficient amounts of human total RNA are not added. However, this would not be expected for blood or semen, where one false negative result was observed for each ( Table 2). Further optimization of assay conditions and/or the development of a human specific RNA quantitation method might serve to reduce the occurrence of false negative results.

### Mock casework

**Figure 3.**
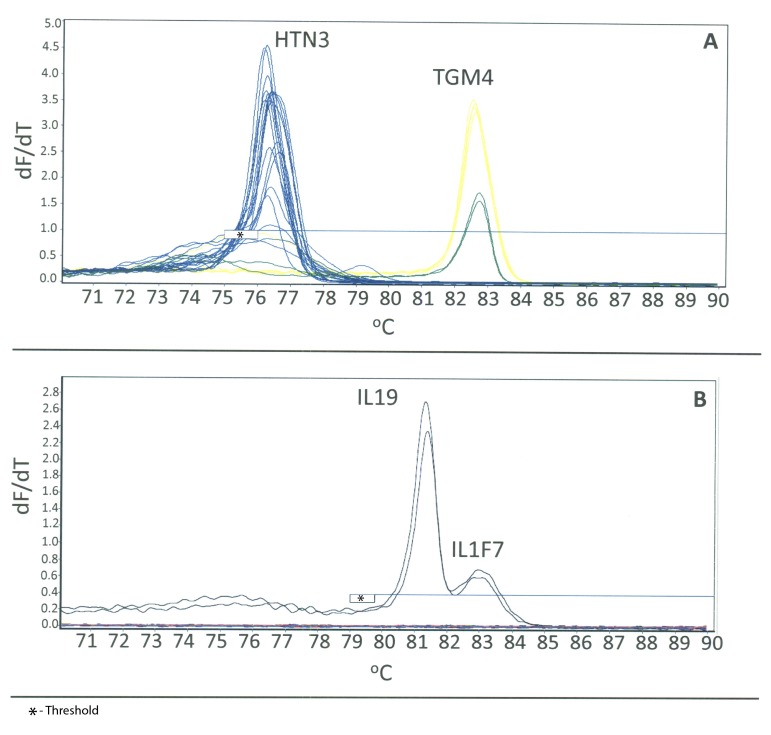
Performance of the saliva/semen and vaginal secretions/skin duplex HRM assays with mock casework samples. **A**) Saliva/Semen Assay. Detection of semen in two different vaginal samples is indicated by the presence of the TGM4 peak. A composite image of known semen and saliva samples (yellow and blue, respectively) and the two vaginal samples (green) in which semen was detected is shown. **B**) Vaginal Secretions/Skin Duplex Assay. Samples were collected from a male finger after digital penetration and from male underwear ~3 hours after intercourse. The detection of the presence of both vaginal secretions and skin is indicated by the presence of the IL19 and IL1F7 peaks in each sample.

**Figure S2.**
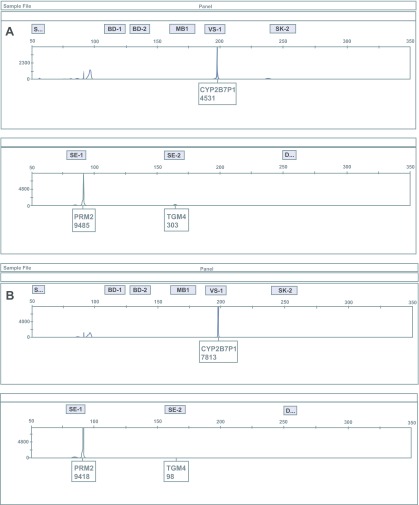
Detection of semen in reportedly single source vaginal samples using a capillary electrophoresis multiple mRNA profiling assay. RT-PCR products from RNA extracted from previously reported single source vaginal secretions samples were amplified in a multiplex reaction using a mRNA profiling assay for body fluid/tissue identification. The multiplex contains mRNA biomarkers for blood, semen, saliva, vaginal secretions, menstrual blood and skin. Shown here are two vaginal samples ( **A** and **B**) in which semen was detected as indicated by the presence of the PRM2 and TGM4 (semen-specific biomarkers). This data is consistent with the detection of semen using the semen-saliva duplex HRM assay. The presence of vaginal secretions is indicated by the presence of CYP2B7P1. The x-axis indicates size in base pairs and the y-axis indicates relative fluorescence units (note: the y-axis scales are different for some panels due to varying signal intensities of the observed products). The grey areas represent the bins for biomarker identification. The biomarker name and relative fluorescence units are displayed.

Evidentiary items of unknown body fluid/tissue origin would be analyzed using each of the three duplexes in order to determine which body fluids or tissues, or combination thereof, are present. We performed such a process during our evaluation of the body fluid duplexes by taking single source samples of known tissue provenance and testing them against the three duplex HRM assays. Interestingly the duplex HRM assays were able to successfully identify contaminated single source samples. A set of reportedly single source vaginal secretions samples were evaluated using each of the three duplex assays to check that no other cross reacting body fluids would be detected with the assay. As can seen from Figure 3A, TGM4 was detected in two of the vaginal samples (melt curves shown in green) indicating the presence of semen. We performed additional testing of these samples using our CE-based mRNA body fluid identification multiplex and were able to confirm the presence of contaminating semen (detection of PRM2 and TGM4) in these two putative single source samples ( Supplementary Figure 2).

The performance of the duplex assays with a limited number of simulated casework samples was evaluated. The mock casework samples were designed to represent possible casework scenarios including digital penetration and sexual assault (vaginal intercourse). The simulated casework samples included (i) a swab of the surface of male fingers after digital vaginal penetration of a female participant and (ii) a swab of the inside of male underwear worn 3 hours after sexual intercourse in order to attempt to detect possible vaginal secretions that might have been transferred from the penis after a sexual assault. An evaluation of potential transfer of vaginal secretions to male underwear worn after intercourse was selected to represent a sexual assault case rather than vaginal swabs since we had previously demonstrated the ability to detect semen in vaginal swabs (described above). For both samples (male finger after digital penetration and the male underwear worn after intercourse) both vaginal secretions (IL19) and skin (IL1F7) were detected ( Figure 3B). These results were consistent with the activity (i.e. behavior) of the participants prior to the collection of samples ^56^.

**Figure 4.**
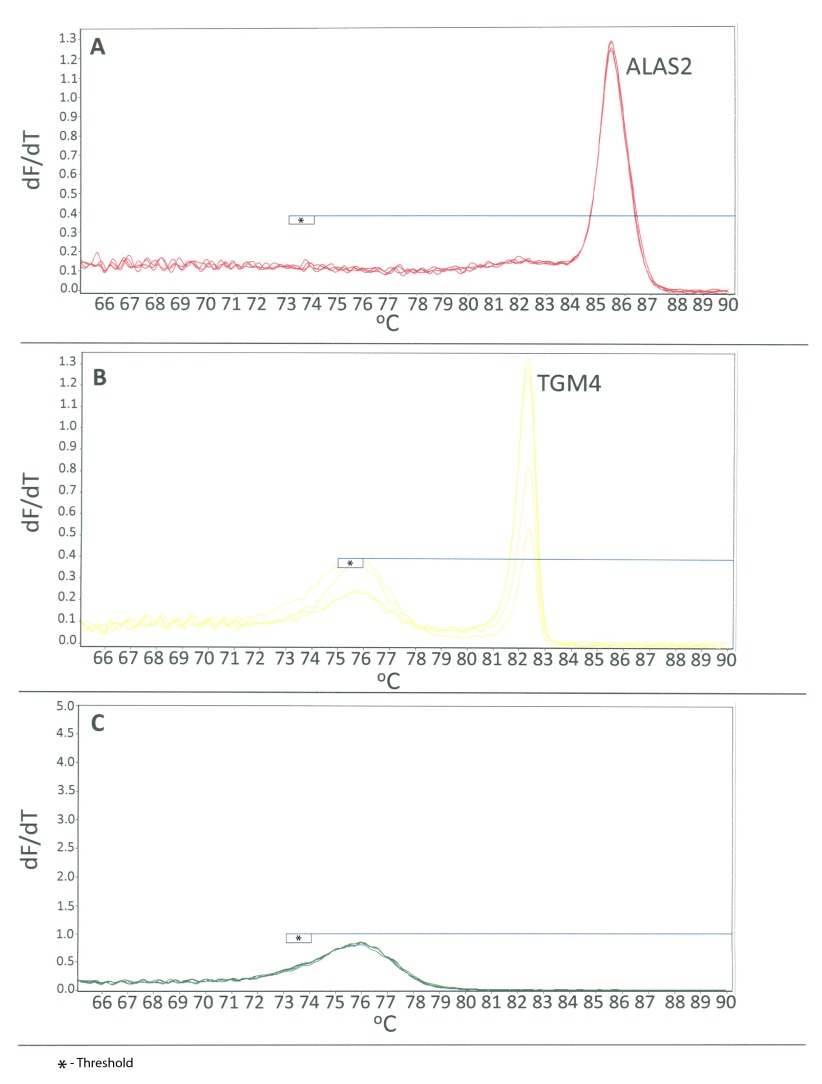
Mixtures. A two-body fluid mixture sample set containing different proportions of the two body fluids was created using a constant amount of blood (50 μl) and decreasing volumes of semen (50 μl, 25 μl, 10 μl, and 5 μl). The admixed samples were co-extracted and the isolated RNA (25 ng) was analyzed using each of the three duplex assays (A – blood/menstrual blood; B – saliva/semen; C – vaginal secretions/skin). Blood and semen were successfully detected, as indicated by the presence of ALAS2 ( **A**) and TGM4 ( **B**) in all four mixture samples.

We evaluated the ability to detect blood and semen in two person body fluid mixtures as an example of a sample admixture type encountered in casework. We prepared blood-semen mixture samples using a constant amount of blood (50 μl) and decreasing amounts of semen (50 μl, 25 μl, 10 μl, and 5 μl). Each admixed sample was evaluated with the three duplex HRM assays ( Figure 4). Blood (ALAS2) and semen (TGM4) were successfully detected in all four admixed samples ( Figure 4). The intensity of the TGM4 peaks varied among the four mixtures samples but did not seem to correlate with the known proportion of semen in the mixture. Thus further work using a more comprehensive sample set is needed to determine whether a quantitative assessment of peak heights correlate to the true marker proportions comprising a mixture.

## Epithelial cell triplex HRM assay

**Figure 5.**
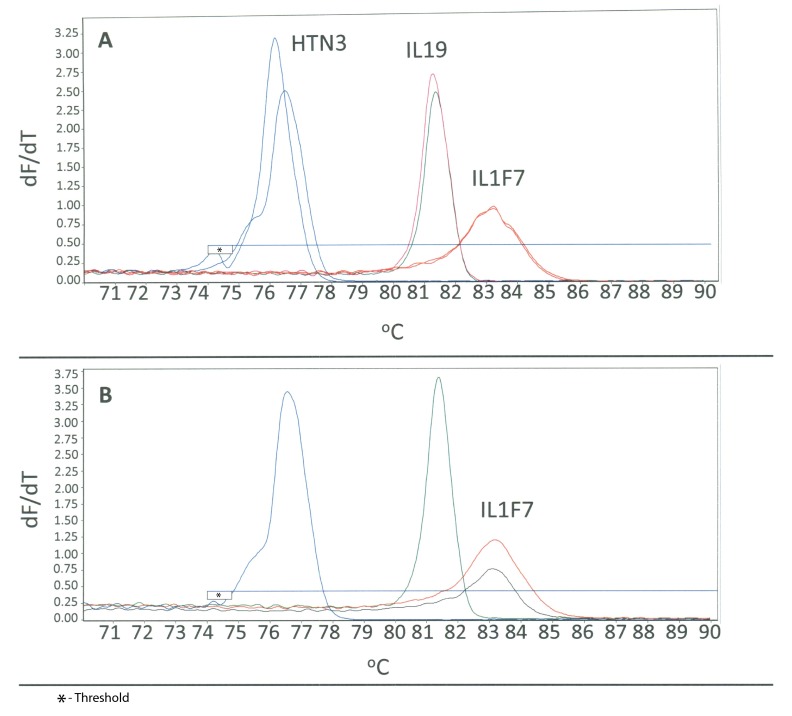
Epithelial cell triplex HRM assay. A Triplex HRM assay was created to permit identification of epithelial-cell containing tissues and fluids (saliva, vaginal secretions and skin). **A**) A body fluid specific marker for each of the three fluids/tissues was incorporated into a single reaction (HTN3 – saliva, IL19 – vaginal secretions, IL1F7 – skin). Each individual sample was analyzed using a single triplex reaction. Two saliva donors (blue), one vaginal secretions (green) and one menstrual blood donor (pink) and two skin donors (orange) are shown overlaid to show the relative locations of each marker in the triplex. **B**) Successful detection of skin from a swab of a computer mouse (black) using the triplex HRM assay is indicated by the presence of IL1F7. A composite image of known saliva, vaginal and skin samples (blue, green and orange, respectively) is shown as a reference.

Since epithelial cells have common functions, namely secretion, selective absorption, protection, trans-cellular transport and detection of sensation ^57^, finding biomarkers capable of unequivocally differentiating and identifying each of the three cell types commonly found in casework (buccal, vaginal and skin epithelia) is challenging. We have recently had success in the identification of highly specific RNA biomarkers for vaginal secretions and skin ^28, 29^ that, in combination with well-characterized saliva markers such as HTN3 or STATH, are capable of differentiating epithelial cells. Encouraged by the success in developing duplex HRM assays, we conceived and formulated a triplex assay for the identification of epithelial cell containing fluids and tissues (i.e. saliva, vaginal secretions and skin). The assay employs the HTN3 (saliva), IL19 (vaginal secretions) and skin (IL1F7) RNA biomarkers ( Figure 5A). The melt curves from single source samples (2 donors for each fluid; for vaginal secretions, one of the two samples is a menstrual blood sample in which vaginal secretions was detected) are shown overlaid in order to demonstrate the non-overlapping location of each marker in the triplex assay. To initially test the performance of this triplex assay with forensic casework type samples, we swabbed the surface of a computer mouse and analyzed it using the triplex assay. The presence of skin was detected on the computer mouse sample as indicated by the presence of IL1F7 ( Figure 5B). This preliminary indication that the sensitivity of the assay might permit the identification of touched objects augurs well for its potential use in casework.

## ‘All body fluids’ hexaplex HRM assay

The availability of a hexaplex assay that would permit a single tube identification of all of the common forensically relevant biological fluids and tissues (blood, semen, saliva, vaginal secretions, menstrual blood and skin) is desirable. The development of such an HRM assay is challenging, however, since the number of biomarkers, or sometimes the choice of biomarker, is dictated by the need to assure non-overlapping *T*_m_ values. Despite differences in amplicon size, the associated *T*_m_ values may be similar or the same depending on the particular amplicon sequences. For example, the inclusion of all biomarkers used in the singleplex, duplex and triplex assays described above into a single hexaplex system would not be possible. The *T*_m_ values of MMP10 overlap with IL19 (vaginal secretions), TGM4 (semen) and IL1F7 (skin). Nevertheless we sought to develop an ‘all body fluids’ hexaplex system using alternative highly specific biomarkers or primer modifications to intentionally alter the *T*_m_ value of the RNA amplicon.

**Figure 6.**
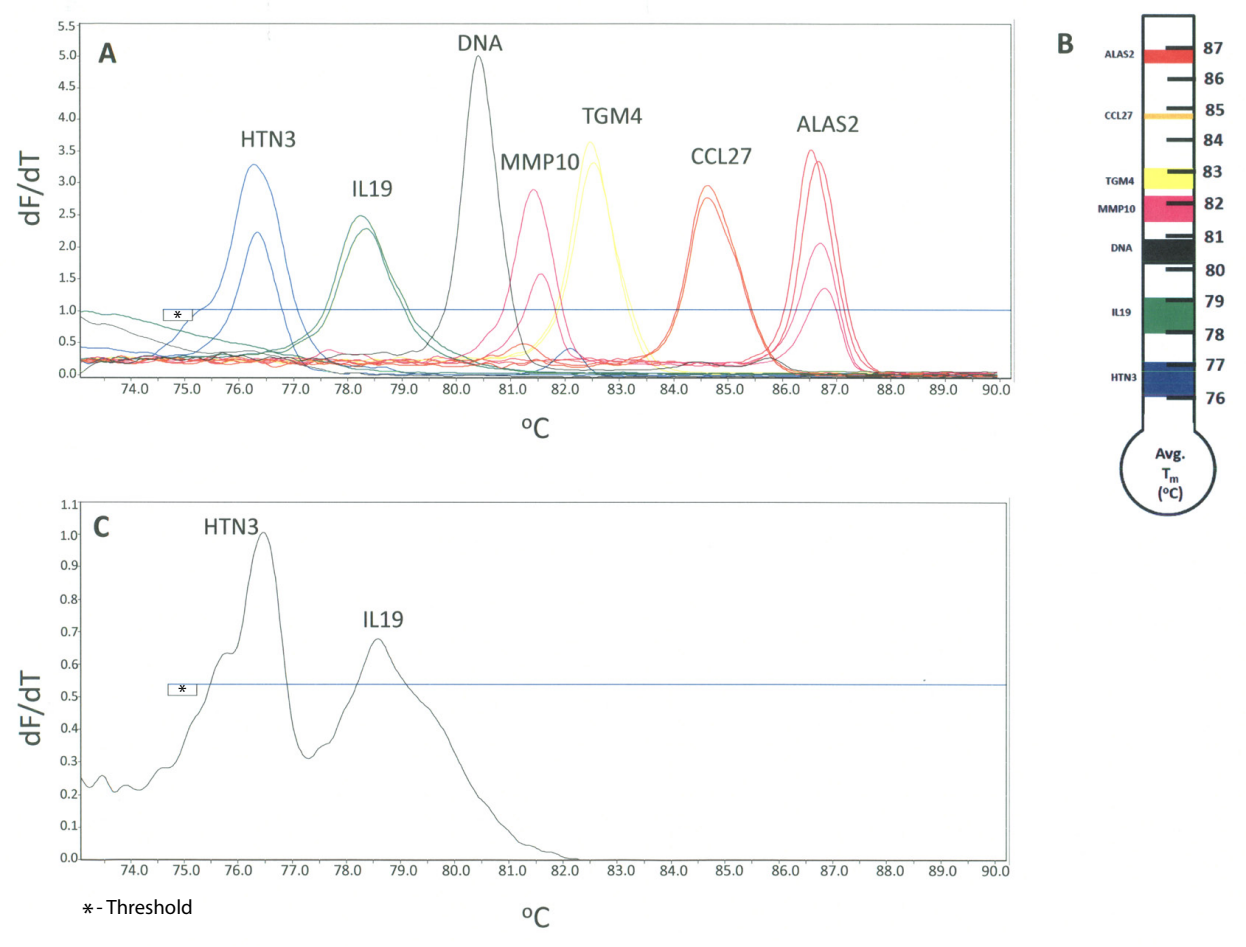
‘All body fluids’ hexaplex HRM body fluid assay. **A**) Derivative melt curve plots for the hexaplex assay is shown: saliva (HTN3, blue), vaginal secretions (IL19, green), menstrual blood (MMP10, pink), semen (TGM4, yellow), skin (CCL27, orange), and blood (ALAS2, red). Two donors of each body fluid or tissue are shown. The melt plots shown are overlaid single source melt plots with all individual samples analyzed using the respective multiplex HRM assays (shown overlaid to demonstrate marker positions within the multiplex assay). The location of a peak originating from contaminating DNA (IL19) is shown in black. **B**) Hexaplex biomarker resolution as represented by the *T*_m_ (°C) range (average *T*_m_ + 3 standard deviations). **C**) Analysis of a saliva-vaginal admixture using the hexaplex. *T*_m_ values of 76.5°C (HTN3) and 78.6°C (IL19) were observed indicating the presence of saliva and vaginal secretions respectively.

The above described singleplex, duplex and triplex assays involved the use of the following markers: ALAS2 (blood), TGM4 (semen), HTN3 (saliva), IL19 (vaginal secretions), MMP10 (menstrual blood) and IL1F7 (skin). Our initial efforts were focused on attempting to include all of these biomarkers in the hexaplex system. However attempts at suitably modifying the IL1F7 primers were unsuccessful and resulted in the requirement for an alternative biomarker for skin. We selected CCL27 as a replacement for IL1F7 based on its *T*_m_ value, sensitivity and specificity ^28^. A CCL27 *T*_m_ value of 84.8°C no longer presented a conflict with MMP10 (81.9°C). However, this was close to the *T*_m_ value of ALAS2 (85.8°C). Sequence modification of the reverse primer for ALAS2 permitted a shift of the observed ALAS2 *T*_m_ value to 86.8°C and therefore resolved any potential overlap with CCL27. The use of an alternative reverse primer sequence for IL19 also resulted in a new observed average *T*_m_ value of 78.6°C and therefore resolved the conflict between MMP10 and IL19. The remaining *T*_m_ conflict was between MMP10 and TGM4. The use of a modified MMP10 forward primer sequence and the inclusion of a “GGGGG” non-template addition to the MMP10 primer set resulted in a sufficient separation of MMP10 and TGM4 *T*_m_ values (~1°C shift of the MMP10 *T*_m_ value). With these modifications a single *T*_m_ value for MMP10 was also observed. With the success of these loci and primer modifications, together with alterations to the PCR cycling parameters, we were able to develop a prototype hexaplex HRM system that was suitable for undergoing a series of preliminary developmental validation studies. The hexaplex system is shown in Figure 6A with single source samples (two donors per body fluid or tissue) overlaid to indicate the location of each biomarker within the hexaplex. All of the incorporated markers are resolvable without any *T*_m_ overlap as indicated by measurement of the average *T*_m_ value and *T*_m_ value range (+ 3 standard deviations) ( Table 3, Figure 6B). A DNA-specific product resulting from amplification with the new IL19 primer set was detected ( Figure 6A, Table 3) but does not interfere with any of the included biomarkers ( *T*_m_ of 80.6°C, between IL19 and MMP10 ranges). The presence of this DNA product can actually be useful in identifying samples that contain significant amounts of contaminating DNA and could therefore be used as an RNA sample quality control check for all sample types, irrespective of body fluid source. Interestingly, similar previously observed DNA products for MMP10 in the duplex assay were not apparent with the hexaplex system.

**Table 3.** Genes and amplicons used in the hexaplex body fluid HRM assay.

Body fluid	Gene	Amplicon size (base pairs)	Amplicon T _m_		Primer sequence (5′–3′)
			°C	Range (± 3SD)	
Saliva	HTN3	134	76.6	76.0 – 77.2	F: GCAAAGAGACATCATGGGTA R: GCCAGTCAAACCTCCATAATC
Vaginal	IL19	109	78.6	78.0 – 79.2	F: AACCACGGTCTCAGGAGATG R: TGACATTTGGGAAGGTGTCC
Menstrual blood	MMP10	178	81.9	81.5 – 82.3	F: GGGGGTGACGTTGGTCACTTCAGCTC R: GGGGGCTGGAGAATGTGAGTGGAGT
Semen	TGM4	164	82.7	82.4 – 83.0	F: ATGGTGTAAAGAGGACATGGTT R: GGGAAATGCAGCAGTCCAG
Skin	CCL27	142	84.9	84.8 – 84.9	F: AGCACTGCCTGCTGTACTCA R: AGATGCTGCGTTGAGCCA
Blood	ALAS2	222	86.8	86.6 – 86.9	F: TGTGTCCGTCTGGTGTAGTA R: GAGTCATTGGCAACAAAGCA
DNA	--		80.6	80.2 – 80.9	NA (product originates from IL19)

*underline indicates non-template sequence addition; report amplicon size includes the non-template sequence additions

We carried out initial performance checks on the hexaplex’s specificity. Single source body fluid and tissue samples (n total = 63; saliva, n = 13; vaginal secretions, n = 9; menstrual blood, n = 4; semen, n = 12; skin, n = 9; blood, n = 16) were tested. The correct biomarker was identified, except for a small number of false negative results with semen (one sample) and saliva (two samples) ( Table 4). Similar to the results of specificity testing for the duplex assays, MMP10 was detected in some of the vaginal secretion samples (3/9, ~33%). Peripheral blood was detected in three of the four menstrual blood samples ( Table 4), which is expected since menstrual blood samples are complex mixtures including peripheral blood and vaginal secretions. Forensic evidentiary items are frequently not single source samples and can contain admixed biological fluids. The results of hexaplex analysis of a saliva-vaginal secretion admixed sample (artificially created mixture in which 25 μl of neat saliva was added to half of a vaginal swab) are shown in Figure 6C. Both saliva (HTN3) and vaginal secretions (IL19) were correctly identified using the hexaplex system.

**Table 4.** ‘All body fluids’ hexaplex HRM assay specificity. The number of single source samples from different individuals in which the marker was successfully detected (numerator) is displayed out of the total number of individuals tested (denominator). The shading reflects the number of positive samples out of the total number tested: white – no detection, light grey 1–74%; dark grey ≥ 75%.

Body fluid/ Tissue (25 ng)	HTN3	IL19	MMP10	TGM4	CCL27	ALAS2
Saliva	**11/13**	0/13	0/13	0/13	0/13	0/13
Vaginal	0/9	**9/9**	**3/9**	0/9	0/9	0/9
Menstrual	0/4	0/4	**4/4**	0/4	0/4	**3/4**
Semen	0/12	0/12	0/12	**11/12**	0/12	0/12
Skin	0/9	0/9	0/9	0/9	**9/9**	0/9
Blood	0/16	0/16	0/16	0/16	0/16	**16/16**

N = 63

Data Sets Tm values for HRM assay specificity Data Set 1. Tm Values for HRM duplex assay specificity This data set contains the individual Tm values for the specificity testing of the vaginal/skin, blood/menstrual and saliva/semen HRM duplex assay specificity presented in Table 2. Individual donors are listed according to body fluid or tissue type with the Tm values (oC) recorded under each biomarker. IL19 = vaginal biomarker, ALAS2 = blood , HTN3 = saliva biomarker, IL1F7 = skin biomarker, MMP10 = menstrual blood, TGM4 = semen biomarker, n.d. = not detected in body fluid of interest, empty cell = no detection
Data Set 2. Tm Values for ‘All Body Fluids’ Hexaplex Assay Specificity This data set contains the individual Tm values for specificity testing of the ‘all body fluids’ hexaplex HRM assay presented in Table 4. Individual donors are listed according to body fluid or tissue type with the observed Tm values recorded under each biomarker. n.d. = not detected in body fluid of interest

**Data Set 1. *T*_m_ Values for HRM duplex assay specificity.**

This data set contains the individual *T*_m_ values for the specificity testing of the vaginal/skin, blood/menstrual and saliva/semen HRM duplex assay specificity presented in Table 2. Individual donors are listed according to body fluid or tissue type with the *T*_m_ values (°C) recorded under each biomarker. IL19 = vaginal biomarker, ALAS2 = blood, HTN3 = saliva biomarker, IL1F7 = skin biomarker, MMP10 = menstrual blood, TGM4 = semen biomarker, n.d. = not detected in body fluid of interest, empty cell = no detection.

**Data Set 2. *T*_m_ Values for ‘All Body Fluids’ Hexaplex Assay Specificity.**

This data set contains the individual *T*_m_ values for specificity testing of the ‘all body fluids’ hexaplex HRM assay presented in Table 4. Individual donors are listed according to body fluid or tissue type with the observed *T*_m_ values recorded under each biomarker. n.d. = not detected in body fluid of interest.

## Discussion

Messenger RNA profiling with a battery of highly specific biomarkers can be used to positively identify all of the commonly found forensically relevant body fluids and tissues. In this work, in order to simplify mRNA profiling assays and to reduce the time and cost of analysis, we have developed a number of prototype multiplex high resolution melt (HRM) assays for the identification of blood, semen, saliva, vaginal secretions, menstrual blood and skin. With respect to critical post-cDNA reagents, the HRM assays require only the use of unlabeled PCR primers and a single intercalating saturating fluorescent dye (Eva Green). In terms of hardware and software a real time instrument with HRM capabilities is required and a number of manufacturers make such instruments. Each body-fluid specific marker can easily be identified by the presence of a distinct melt peak.

The ability to multiplex different combinations of RNA biomarkers as evidenced by the compatibility of the markers in duplex, triplex and hexaplex assays indicates that there exists an opportunity for laboratories to customize HRM assays to suit their specific needs. For example, if the intended use of the HRM assays is to rapidly screen sexual assault evidence, it may be desirable to only incorporate biomarkers for the identification of semen and vaginal secretions or, alternatively, semen, vaginal secretions and saliva. Some may want an epithelial marker assay such as the triplex that can identify and differentiate saliva, vaginal secretions and skin. However, some laboratories may want to utilize HRM assays for the identification of all forensically relevant body fluids.

We recognize that the prototype HRM assays may need to be further optimized (using modified primers, cycling conditions or loci) using a larger and more varied sample set. For example, the semen/saliva duplex assay should be further optimized in order to reduce or eliminate artifacts such as the broad peaks/humps observed in the HTN3 region arising from the TGM4 melt curve. If these artifacts cannot be eliminated it may be necessary to replace TGM4 with another suitable semen marker with a melt curve profile that does not interfere with the interpretation of HTN3 data or to replace HTN3 with another saliva marker that has a higher *T*_m_. It may also be possible to alter the *T*_m_ of the existing markers in the assay by changing the primer location. However, any changes to primer sequences would require additional studies to evaluate primer efficiency and to ensure that no changes to sensitivity or specificity are observed.

The principal advantages of HRM over current mRNA body fluid identification assays that employ capillary electrophoresis (CE) or quantitative RT-PCR (qRT-PCR) platforms are the not insignificant ones of cost and time. Both CE and qRT-PCR platforms require the use of expensive fluorescently labeled primers or probes whereas HRM uses unlabeled primers. CE-based assays require separate amplification and detection steps thus increasing the cost and time required for analysis. Post RNA extraction and cDNA formation, the closed tube HRM assay takes ~ 2 hours to perform which is similar to qRT-PCR assays. However for qRT-PCR assays, only 2 or 3 markers can be included in a single reaction since each marker and internal control requires a different fluorescent dye whereas HRM can multiplex at least 6 markers.

In summary, this proof of principle work describes the design and testing of a number of mRNA HRM assays that, after further validation and optimization, might prove useful in an operational casework setting. The principal advantages of HRM in terms of timeliness and cost may facilitate the technology transfer of mRNA profiling methodology into forensic casework.

## Consent

Body fluids were collected from volunteers using procedures approved by the University’s Institutional Review Board. Informed written consent was obtained from each donor.
