# Supplementary material for: Rapid and inexpensive body fluid identification by RNA profiling-based multiplex High Resolution Melt (HRM) analysis
Source: F1000Res. 2014 Feb 26;2:281. Originally published 2013 Dec 20. [Version 2] doi: 10.12688/f1000research.2-281.v2 (PMC3976110; doi:10.12688/f1000research.2-281.v2)
Supplement: Peer review history

# Peer review: Referee response for version 2

**DOI:** 10.5256/f1000research.3928.r3892

**Published:** 2014-07-28

**Authors:** Gustavo Gutierrez Gonzalez, Chiranjeevi Bodda

**Affiliations:** 1 Vrije Universiteit Brussel, Brussels, Belgium

The authors have satisfactorily addressed all concerns/comments we have initially raised.

We have read this submission. We believe that we have an appropriate level of expertise to confirm that it is of an acceptable scientific standard.

# Peer review: Referee response for version 2

**DOI:** 10.5256/f1000research.3928.r4195

**Published:** 2014-03-19

**Authors:** Titia Sijen

**Affiliations:** 1 Department of Human Biological Traces, Netherlands Forensic Institute, The Hague, Netherlands

I have read this submission. I believe that I have an appropriate level of expertise to confirm that it is of an acceptable scientific standard.

# Peer review: Referee response for version 1

**DOI:** 10.5256/f1000research.3223.r3637

**Published:** 2014-02-19

**Authors:** Titia Sijen

**Affiliations:** 1 Department of Human Biological Traces, Netherlands Forensic Institute, The Hague, Netherlands

Hanson and Ballantyne describe the development of HRM-based single- and multiplex assays for the identification of forensically relevant cell types. These assays are potentially a rapid and relatively inexpensive alternative for mRNA profiling methodologies such as qRT-PCR assays and CE analysis of fluorescently labeled RT-PCR products.

The research is scientifically sound and follows a well-designed stepwise approach. The study is focused at assay development and specificity testing. Known markers were chosen (some more studied such as HTN3, ALAS2, MMP10, TGM4 than others eg IL19, CCL27, IL1F7) that show good specificity with the body fluids tested.

The rationale, results and interpretation of the data are well-described although some figures could be improved (if possible) by using different colors: the use of pink and red to discriminate MMP10 and ALAS2 in Fig2A is somewhat challenging to the eye, and when yellow is used (Fig1D, 2B, 3A, 4B) I found some details quite hard to discern.

The authors state ‘the results demonstrate the potential use of HRM assays for the screening of biological evidence’ and ‘we describe the development and initial performance evaluation of the developed HRM assays’. This raises the question what would be needed before HRM assays would be suited for casework?

In our laboratory, numerous cases have been analyzed using end-point RT-PCR multiplexes on both cell and organ typing detected by CE. These cases have taught us that most/many forensic casework samples will be 1) mixtures of various cell types and 2) of limited stain size. Thus, besides good specificity, sensitivity and the ability to handle (unequal) mixtures are important performance characteristics for RNA assays. The capacity to analyze unequal mixtures will depend on the analysis method. When using CE platforms for instance, high mixture components may result in pull-up signals and artifact peaks that can mask the presence of the minor components or complicate RNA profile interpretation.

Thus, I would like to invite the authors to comment on the expected performance of HRM assays when analyzing unequal mixtures.

Furthermore, as this initial study did not yet assess sensitivity, I am interested in the authors’ thoughts on the sensitivity that they perceive for HRM-based RNA assays.

Overall, I compliment the authors with this study and the novelty they bring to the forensic field.

I have read this submission. I believe that I have an appropriate level of expertise to confirm that it is of an acceptable scientific standard.

# Response

**Published:** 2014-02-24

**Authors:** Jack Ballantyne

**Affiliations:** University of Central Florida, USA

- **"The rationale, results and interpretation of the data are well-described although some figures could be improved (if possible) by using different colors: the use of pink and red to discriminate MMP10 and ALAS2 in Fig2A is somewhat challenging to the eye, and when yellow is used (Fig1D, 2B, 3A, 4B) I found some details quite hard to discern."**

The colors are used to aid visually distinguishing each body fluid. There are always variations between computers and printers, and therefore the original color selected in the instrument software is not always as clearly represented. We also labeled each biomarker so that its location would be clearly indicated on each diagram. We will keep color selection in mind for future publications. 

 

- **"The authors state ‘the results demonstrate the potential use of HRM assays for the screening of biological evidence’ and ‘we describe the development and initial performance evaluation of the developed HRM assays’. This raises the question what would be needed before HRM assays would be suited for casework?"**

A full developmental validation in accordance with SWGDAM guidelines would be required. This would include a more comprehensive evaluation of sensitivity, specificity, reproducibility and performance with mixtures and mock casework samples.

In our laboratory, numerous cases have been analyzed using end-point RT-PCR multiplexes on both cell and organ typing detected by CE. These cases have taught us that most/many forensic casework samples will be 1) mixtures of various cell types and 2) of limited stain size. Thus, besides good specificity, sensitivity and the ability to handle (unequal) mixtures are important performance characteristics for RNA assays. The capacity to analyze unequal mixtures will depend on the analysis method. When using CE platforms for instance, high mixture components may result in pull-up signals and artifact peaks that can mask the presence of the minor components or complicate RNA profile interpretation.

 

- **"Thus, I would like to invite the authors to comment on the expected performance of HRM assays when analyzing unequal mixtures**."

A blood-semen mixture, with decreasing amounts of semen in each mixture, was evaluated using the duplex assays. Both body fluids were correctly identified in each of these mixtures. Successful detection of both components of a saliva-vaginal admixed sample was also observed for the hexaplex assay. While these were not large-scale comprehensive mixture studies, we are hopeful that the HRM assays will permit the ability to identify all components in admixed body fluid samples. Similar to the experience of the referee with CE based assays, It is likely that some mixture types will be challenging to define because of the particular mixture composition or because one or more of the components are present as a minor fraction. In our experience with CE as well, highly abundant biomarkers tend to dominate detection and can mask the presence of a minor component. It will be interesting to see with future studies whether the use of the HRM assays will permit an improved detection of minor component fluids or tissues. So while more comprehensive mixtures studies are needed (and will be included as part of a full developmental validation), we are hopeful that the successful initial results presage the efficacy of HRM assays to perform reasonably well with mixtures.  [See also the response to the first referee report].

 

- **"Furthermore, as this initial study did not yet assess sensitivity, I am interested in the authors’ thoughts on the sensitivity that they perceive for HRM-based RNA assays."**

We performed initial sensitivity studies for each of the HRM duplex assays. The sensitivity of each of the duplex assays was evaluated by testing a range of input total RNA, from 15pg to 100ng (15pg, 25pg, 50pg, 100pg, 250pg, 500pg. 1mg, 5ng, 10ng, 25ng, 50ng and 100ng). For the blood/menstrual blood duplex assay, both biomarkers (ALAS2 and MMP10) were detected using picogram quantities of input total RNA (consistently detected using 250 pg or more; variable below 250pg (positive results for both biomarkers with 15 and 50pg input, but not 25 and 100pg)). For the saliva/semen assay, both biomarkers (HTN3 and TGM4) were detected using as little as 25pg input. Using 15pg input, HTN3 was also detected but not TGM4. Therefore the sensitivities of these two duplex assays appear to be highly sensitive, requiring only picogram levels of input total RNA. Additionally, it should be noted that the total RNA input amounts listed represent the amount used in the RT reaction. Detection of product in the real time assays is performed with 1/10 ^th^ of the RT input amount (2ml cDNA/20ml reaction). For the vaginal secretions/skin duplex assay, 5ng of input RNA (still only 500pg for detection) was required for IL19. Skin was successfully detected with as little as 100pg total RNA RT input. Since only one donor was tested for this initial sensitivity work, the actual sensitivity limits need to be determined with additional testing. For vaginal secretions, since no human specific RNA quantitation method is currently available, there is always a potential for the obtained RNA quantitation value to be artificially higher due to the presence of endogenous bacteria. Therefore, the total RNA input used for these samples may not be fully accurate which will then affect the sensitivity results. But overall, these assays appear to require picogram levels of input total RNA, which would make them suitable for use with forensic, samples.

An initial sensitivity check for the hexaplex assay was also performed (again only one donor per fluid; 5 pg to 25 ng range of total RNA input into the RT reaction). The body fluids were detected down to varying lower input limits: blood (ALAS2) - 25 pg; menstrual blood (MMP10) - 250 pg; skin (CCL27) - 1ng; semen - 5ng; vaginal secretions – consistently with 1 ng or more input (variable detection in the 5pg to 1ng range); saliva (HTN3) – not determined at this time.

While we performed this preliminary sensitivity evaluation, it was not included in the manuscript since only one donor was evaluated. More comprehensive sensitivity studies, particularly for the hexaplex system, will be included in a future publication describing a comprehensive testing and evaluation of the HRM assay.

# Peer review: Referee response for version 1

**DOI:** 10.5256/f1000research.3223.r2871

**Published:** 2014-01-09

**Authors:** Gustavo Gutierrez Gonzalez, Chiranjeevi Bodda

**Affiliations:** 1 Vrije Universiteit Brussel, Brussels, Belgium

Hanson & Ballantyne report in this article a novel use for High Resolution Melt analysis of RNA samples in order to identify single or mixed human body fluids (and tissues), namely vaginal secretions, skin, blood, menstrual blood, saliva, or semen using a series of molecular biomarkers: IL19, IL1F7 or CCL27, ALAS2, MMP10, HTN3, and TGM4, respectively. Identification of these body fluids/tissues can be of crucial importance in forensic sciences and criminal cases. The methodology appears to be quite specific and versatile, and according to the authors, less expensive and time-consuming compared to similar mRNA-based platforms such as quantitative real-time polymerase chain reaction or capillary electrophoresis. Remarkably, the technique can allow the identification of up to 6 body fluids in one single reaction (i.e., hexaplex assay).

The paper is very well written, scientifically sound and their results are very clear and adequately interpreted and discussed. I support its approval in the journal F1000 Research. I also have several comments to be eventually addressed:

 

1. Would there be a way to differentiate between blood and menstrual blood samples? Would that be something important to achieve?
2. Have the authors used (or planned to use) this technique with samples such as sweat, urine, or hair? Additionally, could the technique differentiate human from animal samples (using the same biomarkers)?
3. Can the authors give a little bit more information about the size of the amplicons generated by PCR for the different biomarkers employed and also about the design of the primers employed?
4. How reproducible is the assay for one sample coming from an individual?
5. We noticed that when you compare tables 2 and 4, there is a significant improvement of the specificity using the IL19 marker in the hexaplex assay. As mentioned by the authors, they have used a different reverse primer for IL19 in the hexaplex assay. We wonder whether this new reverse primer can also improve the specificity for IL19 to differentiate vaginal secretions from menstrual blood also in single, duplex, or triplex analyses.
6. Regarding the possible amplification of MMP3 by MMP10 primers in the menstrual blood samples under certain experimental conditions. I think it could be important to test that this is indeed the case; maybe by designing MMP3 specific primers that confirm this particular peak or eventually sequencing the products that were generated.
7. Finally, I got the impression from the experiments in which the authors mixed blood with decreasing amounts of semen that the technique does not necessarily reflect quantitative trends. Is that indeed the case? Could quantitation be ultimately important in a forensic case?

We have read this submission. We believe that we have an appropriate level of expertise to confirm that it is of an acceptable scientific standard.

# Response

**Published:** 2014-01-29

**Authors:** Jack Ballantyne

**Affiliations:** University of Central Florida, USA

- **Would there be a way to differentiate between blood and menstrual blood samples? Would that be something important to achieve?**

The described hexaplex assay has a biomarker for both blood and menstrual blood. Therefore they will be distinguished using this assay. However, menstrual blood will also contain varying amounts of peripheral blood and therefore both the blood (ALAS2) and menstrual blood (MMP10) biomarkers may be present in menstrual blood samples. If the sample is strictly peripheral blood, only the ALAS2 (blood) biomarker would be present and the menstrual blood (MMP10) marker would be absent. This can be important in some cases where intimate contact is implied or suggested based on the circumstances of the case and a suspect argues that it is blood from the nose of the victim, for example, during an assault but not a sexual assault. The identification of menstrual blood as the source would indicate more intimate contact. Additionally during sexual assault investigations, peripheral blood could indicate that trauma had taken place to the female reproductive system, and therefore a determination that the blood present is not simply menstrual blood from normal menstruation could be probative.

 

- **Have the authors used (or planned to use) this technique with samples such as sweat, urine, or hair? Additionally, could the technique differentiate human from animal samples (using the same biomarkers)?**

We have not yet used this technique with other types of samples such as sweat, urine or hair. The HRM approach described here could certainly be utilized for the identification of other forensically relevant materials if suitable biomarkers were identified. This could also include internal organs and tissues tissues such as brain, heart, etc. This work was focused on those body fluids and tissues commonly found at the crime scene, but the approach could certainly be used for other fluids and tissues in the future.

 

- **Can the authors give a little bit more information about the size of the amplicons generated by PCR for the different biomarkers employed and also about the design of the primers employed?**

All of the primers were designed using Primer 3 software. Primers were designed to either span exon-exon boundaries (no DNA amplification) or designed to be in separate exons (so that the DNA product size would be different than the RNA product size due to the inclusion of an intron). Tables 1 and 3 have been updated to include amplicon size and also the exon location of the primers.

 

- **How reproducible is the assay for one sample coming from an individual?**

The assay is highly reproducible within an individual. Typically in a single experiment the Tm curves from the same individual are exactly superimposable upon one another The donors used in this study were also used repeatedly throughout testing, with both separate RNA extractions and multiple RT-PCR’s from the same individual extract being employed. No significant differences in Tm were observed.

 

- **We noticed that when you compare tables 2 and 4, there is a significant improvement of the specificity using the IL19 marker in the hexaplex assay. As mentioned by the authors, they have used a different reverse primer for IL19 in the hexaplex assay. We wonder whether this new reverse primer can also improve the specificity for IL19 to differentiate vaginal secretions from menstrual blood also in single, duplex, or triplex analyses.**

Menstrual blood samples will, by nature of the source of the material, contain varying amounts of vaginal secretions. Therefore IL19 in a singleplex assay still will never provide sufficient resolution to distinguish between a vaginal and menstrual sample, as both could contain vaginal secretions. While every menstrual blood sample may not contain sufficient levels of vaginal material to result in IL19 detection, vaginal secretions will still be present in such samples to a varying extent. Therefore a menstrual blood-specific biomarker is typically needed to definitively identify the presence of menstrual blood. A duplex reaction with IL19 and MMP10 for example would permit a determination of vaginal secretions and menstrual blood. Vaginal secretions only samples should have only IL19, whereas menstrual blood samples could contain only MMP10 or a mixture of MMP10 and IL19.

- **Regarding the possible amplification of MMP3 by MMP10 primers in the menstrual blood samples under certain experimental conditions. I think it could be important to test that this is indeed the case; maybe by designing MMP3 specific primers that confirm this particular peak or eventually sequencing the products that were generated.**

It is certainly of interest to determine whether the additional product is in fact MMP3 and to determine if specific MMP10 and MMP3 primer sets could be distinguished. This will be the subject of future work, although it is important to note that this uncertainty does not interfere with the accurate interpretation of the body fluid results using the current assay.  

 

- **Finally, I got the impression from the experiments in which the authors mixed blood with decreasing amounts of semen that the technique does not necessarily reflect quantitative trends. Is that indeed the case? Could quantitation be ultimately important in a forensic case?**

In our evaluation so far of data from the HRM assays, the height of the melt peak, somewhat surprisingly, does not appear to correlate with the amount of analyte.  More in depth studies need to be performed in order to determine possible reasons for this. It should be noted that the melt peak is the first derivative of fluorescence with temperature. It might be the case that other transforms of the data other than the first derivative might prove to be better correlated with analyte input.

The ability to make a relative quantitative assessment of the amount of each body fluid present in an unknown sample would be useful. The RNA quantitation method we employ, namely the binding of the fluorescent dye RiboGreen, provides an overall assessment of the amount of RNA present, but cannot provide quantitative information regarding the amount of each fluid present if the sample contains more than one body fluid. While a quantitative assessment is not critical for a determination of the presence or absence of a body fluid, a quantitative assessment could be provide important probative information for admixed body fluid samples.
